# CT findings of gastric and intestinal anisakiasis

**DOI:** 10.1007/s00261-014-0075-3

**Published:** 2014-01-18

**Authors:** Eisuke Shibata, Takuya Ueda, Gensuke Akaike, Yukihisa Saida

**Affiliations:** 1 Department of Radiology, Tokyo Metropolitan Police Hospital, 4-22-1 Nakano, Nakano-ku, Tokyo, 164-8541 Japan; 2 Department of Radiology, Graduate School of Medicine, The University of Tokyo, 7-3-1 Hongo, Bunkyo-ku, Tokyo, 113-8655 Japan; 3 Department of Radiology, St. Luke’s International Hospital, 9-1 Akashi-cho, Chuo-ku, Tokyo, 104-5860 Japan

**Keywords:** Anisakis larvae, Gastric anisakiasis, Intestinal anisakiasis, Computed tomography

## Abstract

**Purpose:**

To illustrate the CT findings of gastrointestinal anisakiasis.

**Subjects and methods:**

The Institutional Review Board approving this retrospective study waived the requirement for informed consent. Review of our emergency department’s clinical records from September 2008 to January 2012 identified 41 consecutive patients who were diagnosed with gastrointestinal anisakiasis. 20 patients were diagnosed with gastric anisakiasis with endoscopically proven Anisakis larvae, and 21 patients were diagnosed with intestinal anisakiasis with positive test results for anti-anisakidae antibody and the presence of intestinal lesions on CT. Two radiologists retrospectively assessed the CT findings.

**Results:**

The mean time delay from raw fish ingestion to symptom onset was 5.2 h (range 0.5–24 h) in gastric anisakiasis and 39 h (range 12–120 h) in intestinal anisakiasis. Gastric anisakiasis showed marked submucosal edema of the gastric wall (20/20 patients, 100%), increased attenuation of adjacent fat (19/20, 95%), and ascites (14/20, 70%) on CT. Intestinal anisakiasis showed marked submucosal edema of the intestine (21/21 patients, 100%) without showing complete intraluminal occlusion, ascites (21/21, 100%), increased attenuation of adjacent fat (19/21, 90%), and fluid collection in the distal segment of the constricted small intestine (13/21, 62%) on CT.

**Conclusion:**

Severe submucosal edema with ascites is a characteristic finding of gastrointestinal anisakiasis when compared with other forms of gastroenteritis. When CT shows the typical findings of gastrointestinal anisakiasis, radiologists may suggest the possibility of clinically undiagnosed anisakiasis, especially in intestinal anisakiasis as the diagnosis is sometimes difficult due to the long interval between food intake and symptom onset.

Anisakiasis is a human parasitic infection of the gastrointestinal tract caused by the consumption of raw or undercooked seafood, such as fish or squid containing the Anisakis nematode larvae [1]. Since the first case of anisakiasis was published by van Thiel [2], it has been frequently reported in areas of the world where fish is consumed raw or lightly pickled. As the recent trend of cultural globalization of food habits increases opportunities to eat raw or undercooked seafood, the risk of suffering anisakiasis may also increase worldwide.

In anisakiasis, humans are the accidental host of the parasite. After foods containing the Anisakis larvae are ingested, the larvae invade the gastric and intestinal walls. Gastrointestinal invasion causes direct tissue damage and an allergic reaction of the gastrointestinal wall. The damage then develops an eosinophilic granuloma, ulcer, or perforation of the gastrointestinal wall [3, 4]. Clinical presentation differs according to the site of involvement. Gastric anisakiasis is more common than small or large intestinal anisakiasis [4]. Patients with gastric involvement of anisakiasis typically present with the abrupt onset of abdominal pain, nausea, sometimes vomiting or diarrhea, with signs of peritoneal irritation and incomplete ileus of the small intestine [3]. In addition to the direct damage to the involved intestine, an acute allergic reaction may occur accompanied by an immunoglobulin IgE-mediated systemic allergic reaction [5]. Symptoms of the allergic reaction in anisakiasis range from urticaria and angioedema to life-threatening anaphylactic shock associated with gastrointestinal symptoms [6]. The treatment of gastric anisakiasis is either endoscopic removal of the parasites or conservative management. Intestinal anisakiasis is generally treated with conservative management.

In gastric anisakiasis, the Anisakis larvae are frequently found on gastric endoscopy. In intestinal anisakiasis, the diagnosis is commonly made with a combination of the positive results for anti-anisakidae antibody and the presence of the intestinal lesions on CT. Elevated serum levels of both IgG and IgA antibodies to the Anisakis larvae prove the infection, but this does not identify the exact location of the infection. Although there were many case reports of gastrointestinal anisakiasis focusing on clinicopathological features, the CT findings of gastrointestinal anisakiasis were not fully understood [7]. The purpose of this study is to investigate the radiological findings of gastrointestinal anisakiasis and to discuss the differential diagnosis in acute abdominal diseases.

## Materials and methods

### Patients

The Institutional Review Board approved this retrospective study and waived the requirement to obtain informed consent from patients. Medical records of the emergency department of our institution between March 2008 and February 2012 were reviewed. A total of 41 consecutive patients who were diagnosed with gastrointestinal anisakiasis (20 patients with gastric anisakiasis and 21 patients with intestinal anisakiasis) were enrolled in the study. Medical charts were reviewed for the following clinical features: symptoms, a clinical history of intake of raw or undercooked fish, the interval time from the intake of foods to the onset of abdominal symptoms, and the method of diagnostic tests.

### CT protocol

Abdominal CT examinations were performed either on 16-MDCT scanners (Bright Speed Series, GE Healthcare) or 64-MDCT scanners (Aquilion, Toshiba Medical Systems) in a single institution. It is the standard practice in our emergency department to perform non-contrast-enhanced CT followed by contrast-enhanced CT for all acute abdomen patients in order to detect hemorrhage and calcification. Contrast-enhanced CTs were performed in all patients, unless it was contraindicated, with the administration of contrast media after a non-contrast enhanced scan (80–100 ml of 300–320 ‰ of non-ionic contrast media depending on the patient’s body weight). The contrast-enhanced images were obtained at 90 s after intravenous administration of contrast media injected at the rate of 3.5 ml/s. Reconstructed images of 5-mm slice thickness were used for assessment.

### CT evaluation

Two radiologists (one with 3 years of experience in general radiology and one with 40 years of experience in abdominal imaging) evaluated the contrast-enhanced CT images of gastric and intestinal anisakiasis, and a consensus was reached.

Based on the radiological findings of previous case reports [7, 8] and the clinical experience of our institution, the radiological features of gastric and intestinal anisakiasis were assessed for the following findings: the presence of submucosal edema of the stomach and intestine, fat infiltration of the mesentery around the involved stomach and intestine, dilatation, and fluid collection of the involved stomach and intestine, abdominal lymphadenopathy, and the presence of ascites. The duodenum and small bowel were classified as “dilated” when the bowel lumen was >3 cm, measured from one outer wall to the opposite outer wall. Fluid collection in the involved duodenum and the small bowel was defined as the condition when the dilated gastrointestinal tract was filled with fluid along a length of 5 cm. Lymph nodes over 10 mm in diameter in the short axis were considered positive for lymphadenopathy.

## Results

### Clinical features

Clinical features of the patients are summarized in Table 1. The mean age of the patients was 45.0 years (range 19–70 years). Diagnoses at the time of medical interview before CT examination are listed in Table 2. 26 patients received an incorrect diagnosis before CT examination. At the initial medical review, all 20 patients (100%) who were diagnosed with gastric anisakiasis provided an evident clinical history of intake of raw or undercooked fish, such as sushi and sashimi, at the initial medical interview, 0.5–24 h before the onset of the abdominal symptoms. All 21 patients (100%) who were diagnosed with intestinal anisakiasis were not correctly diagnosed at the time of the medical interview until a clear clinical history of intake of raw or undercooked fish was provided. After CT examination suggested the possibility of intestinal anisakiasis, 19 out of the 21 patients finally confirmed the clinical history of intake of raw or undercooked fish between 12 h and 5 days before the onset of the abdominal symptoms (Table 1).

**Table 1:** Patient information of gastric and intestinal anisakiasis

	Gastric anisakiasis	Intestinal anisakiasis
Patient number	20	21
Gender male:female	11:9	17:4
Mean age (range)	40.6 (27–65)	49.1 (19–70)
Onset of symptoms after eating contaminated food^a^		
<12 h	16	1
12–24 h	4	9
24–48 h	0	8
<48 h	0	1 (5 days)
Mean (range)	9.1 h	1.6 days

^a^We excluded two patients with intestinal anisakiasis, because patients did not realize when they had taken contaminated raw fishes

**Table 2:** Clinical dagnosis before CT examinations

Clinical diagnosis before CT examination	*n* (%)
Gastric anisakiasis (*n* = 20)	
Gastric anisakiasis	14 (70)
Cholecystitis and/or cholangitis	2 (10)
Pancreatitis	1 (5)
Diverticulitis of the colon	1 (5)
Unknown	2 (10)
Intestinal anisakiasis (*n* = 21)	
Ileus	9 (43)
Diverticulitis of the colon	2 (10)
Appendicitis	2 (10)
Intestinal anisakiasis	1 (5)
Perforation of the gastrointestinal tract	1 (5)
Aortic dissection	1 (5)
Unknown	5 (24)

### CT findings

Table 3 summarizes the CT findings of gastric and intestinal anisakiasis.

**Table 3:** CT findings of gastric and intestinal anisakiasis

CT findings	*n* (%)
Gastric anisakiasis (*n* = 20)	
Severe submucosal edema of the gastric wall	20 (100)
Fat infiltration around the stomach or retroperitoneum	19 (95)
Ascites	14 (70)
Dilatation and fluid collection in the duodenum	14 (70)
Enlargement of lymph nodes	7 (35)
Intestinal anisakiasis (*n* = 21)	
Localized submucosal edema of the intestinal wall	21 (100)
Ascites	21 (100)
Fat infiltration of the mesentery	19 (90)
Fluid collection in the distal intestinal tract	13 (62)
Enlargement of lymph node in the mesentery	11 (52)

**Fig. 1:**
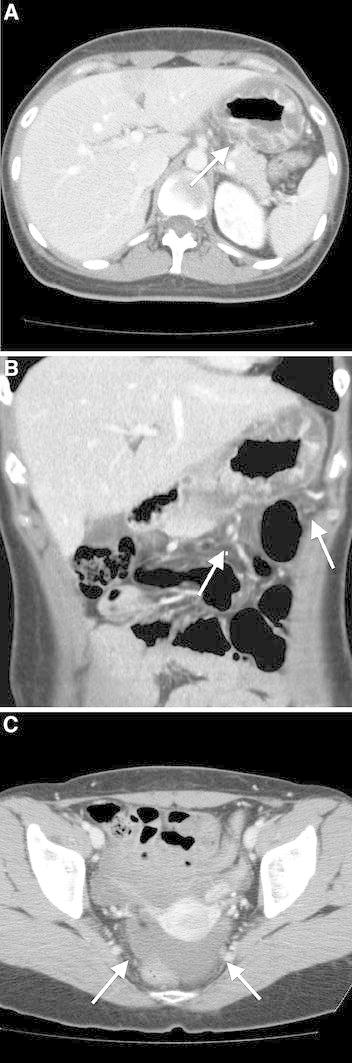
Gastric anisakiasis in a 32-year-old woman who presented with severe epigastric pain and a history of eating raw fish 1 day before symptom onset. **A** The CT image shows the severe mucosal edema of the whole gastric wall (*arrow*). **B** The coronal reconstructed image shows the edematous change of the stomach. Fat infiltration was also seen around the stomach (*arrow*). **C** A small amount of ascites was present in the pelvis (*arrow*).

All 20 patients who were diagnosed with gastric anisakiasis demonstrated significant submucosal edema (Fig. 1). 10 of the 20 patients (50%) showed submucosal edema in the entire gastric wall, 9/20 patients (45%) in the gastric body or antrum of the stomach, and 1/20 patient (5%) in the cardia of the stomach. Fat infiltration around the stomach was observed in 19/20 patients (95%). Ascites was observed in 14/20 patients (70%). 14 out of 20 patients (70 %) had fluid content in the dilated duodenum without severe submucosal edema. Lymphadenopathy was observed in 7 patients (35%).

**Fig. 2:**
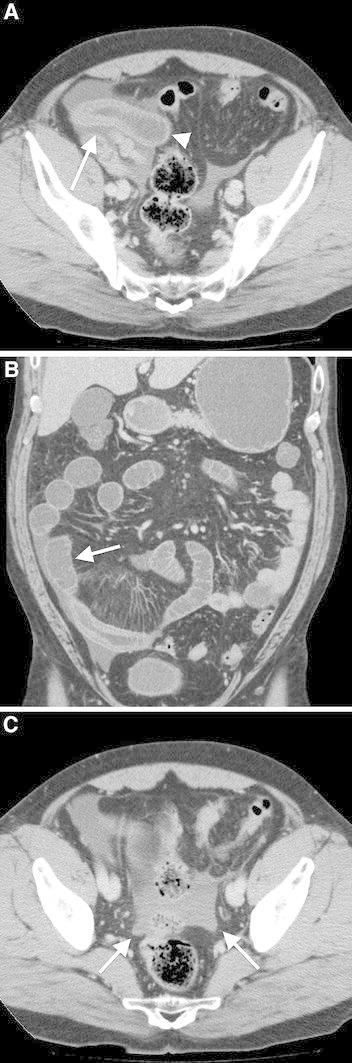
Intestinal anisakiasis in a 50-year-old man who presented with severe abdominal pain and a clinical history of eating raw fish 2 days before symptom onset. **A** CT shows severe, regional, submucosal edema of the small intestine (*arrow*). The proximal small bowel is dilated and fluid-filled (*arrow head*). **B** The distal small intestine of the involved intestinal tract is dilated with fluid collection (*arrow*). **C** The patient had ascites in the pelvis (*arrow*).

All 21 patients (100%) who were diagnosed with intestinal anisakiasis had severe submucosal edema (Fig.2). 20 out of 21 patients (95%) showed dilated and fluid-filled small bowel loops in the proximal of the intestine that showed severe submucosal edema. 13 out of 21 patients (62%) also showed fluid collection in the distal part of the involved small intestine. 19 out of 21 patients (90%) showed increased CT attenuation of the mesenteric fat around the involved intestine. 11 out of 21 patients (52%) showed mesenteric lymphadenopathy.

## Discussion

CT findings of gastrointestinal anisakiasis are summarized in three characteristic observations: 1. severe submucosal edema of the involved gastrointestinal area, 2. ascites and fluid collection in the intestinal distal to the involved area, and 3. lack of obstruction of the bowel at the site of severe mucosal edema.

The pathological characteristics of anisakiasis consist of a combination of two different mechanisms: direct tissue damage and allergic reaction by Anisakis larvae [3, 4]. Direct tissue damage due to the invasion of Anisakis larvae involves development of an eosinophilic granuloma or perforation [9]. Takei et al. [10] reported the pathological findings of intestinal anisakiasis: the small intestine showed transmural edema, congestion, and diffuse infiltration of neutrophils as well as eosinophils. The typical inflammatory infiltrate, rich in eosinophils, is seen as an eosinophilic granuloma or an eosinophilic abscess. Those pathophysiological findings are directly reflected on the characteristic CT findings in our study.

The clinical differential diagnosis of gastric anisakiasis includes acute gastric mucosal lesion (AGML) or gastric ulcer, eosinophilic gastritis, and caustic ingestion. The clinical differential diagnosis of intestinal anisakiasis includes bacterial or viral enteritis, eosinophilic gastroenteritis, and Crohn’s disease.

The clinical diagnosis of gastric anisakiasis is usually easy as the patients commonly provide a typical clinical history and the Anisakis larvae are often proved by endoscopic examination. Therefore, the role of the CT in gastric anisakiasis is to rule out other fatal abdominal diseases that show similar clinical symptoms. Intestinal anisakiasis is often clinically under-recognized due to the long interval (commonly 1 week) from the intake of contaminated food to the onset of symptoms. A specific serum blood test for anisakiasis is not routinely undertaken unless it is suspected based on the clinical history. In fact, many patients were clinically diagnosed with other abdominal diseases before the CT findings suggested the possibility of anisakiasis in our study. Therefore, CT is a useful tool to suggest the possibility of the under-recognized diagnosis of intestinal anisakiasis to clinicians. In this study, anisakiasis is more frequent in men than in women. Yasunaga et al. [7] also reported a male predominance of anisakiasis. Although the reason for the male predominance of anisakiasis remains unknown, this may be because men consume raw fish or sushi more frequently based on food habits.

Our study has some limitations. First, a small number of patients were included in this study, although our study includes a larger number of patients than previous studies. Second, our study was performed retrospectively. Third, the patient selection in our study may have been biased. Since the antigen test was performed only for the patients who were suspected of having intestinal anisakiasis, the patients who were not suspected of having intestinal anisakiasis by CT scan may have been missed in this study. Finally, we did not compare the CT findings of gastrointestinal anisakiasis with those of other abdominal diseases.

In conclusion, our study demonstrated characteristic CT findings of gastric and intestinal anisakiasis: severe submucosal edema of the involved gastrointestinal tract, fluid collection, and ascites without showing severe bowel obstruction. An understanding of the characteristic CT findings of gastrointestinal anisakiasis is useful for suggesting the possibility of this clinically under-recognized diagnosis to clinicians.
